# Long-term psychological and functional outcomes after hepatitis C eradication with direct-acting antivirals: an 80-month follow-up study

**DOI:** 10.3389/fpsyt.2026.1768493

**Published:** 2026-05-25

**Authors:** Salvatore Cipolla, Pierluigi Catapano, Maria Chiara Della Corte, Daniele De Francesco, Antonio Volpicelli, Iolanda Cafarella, Filomena Boccia, Lorenzo Bertolino, Emanuele Durante-Mangoni, Rosa Zampino, Mario Luciano

**Affiliations:** 1 Department of Psychiatry, University of Campania “Luigi Vanvitelli”, Naples, Italy; 2 Department of Advanced Medical and Surgical Science, University of Campania “Luigi Vanvitelli”, Naples, Italy; 3 Internal and Transplant Medicine, AORN dei Colli, Monaldi Hospital, Naples, Italy; 4 Department of Precision Medicine, University of Campania “Luigi Vanvitelli”, Naples, Italy

**Keywords:** coping strategies, direct-acting antivirals (DAAs), hepatitis C virus (HCV), long-term follow-up, mental health, quality of life

## Abstract

**Introduction:**

Direct-acting antivirals (DAAs) have dramatically changed hepatitis C virus (HCV) treatment, by achieving high virological cure rates and a reduced percentage of adverse events compared with previous treatments. Despite this, long-term psychiatric and quality-of-life (QoL) outcomes after viral eradication remain insufficiently understood. This study provides an 80-month follow-up of a previously evaluated cohort.

**Methods:**

Of the original 62 patients, 24 (38.7%) were reassessed approximately 80 months after DAA initiation. Psychopathological symptoms (HAM-D, HAM-A, SCL-90-R), coping strategies (COPE), and QoL (SF-36) were evaluated and compared with baseline (T0). Patients were stratified by psychiatric history (Group P vs. Group NP). Non-parametric tests and exploratory correlations and regressions were performed.

**Results:**

In Group P, depressive and anxiety symptoms significantly improved from T0 to follow-up (HAM-D: 16 vs. 3, p < 0.01; HAM-A: 15 vs. 4, p < 0.01), with additional reductions in SCL-90-R Interpersonal Sensitivity, Paranoid Ideation, and Psychoticism (all p < 0.05). Group NP showed stable psychological profiles, reflecting the maintenance of improvements already observed at T1, with significant reductions in HAM-D (7 vs. 4, p < 0.01) and HAM-A (8 vs. 4, p < 0.001). Both groups demonstrated a significant decline in SF-36 Physical Component Summary (Group P: p < 0.05; Group NP: p < 0.01). No between-group differences were detected at follow-up. Avoidant coping and psychiatric history were significant negative predictors of long-term anxiety change.

**Conclusions:**

Seven years after HCV eradication, psychological well-being remains stable or improved, while physical QoL declines. DAAs demonstrate sustained long-term psychiatric safety, underscoring the need for integrated medical–psychological follow-up in HCV survivors.

## 1 Introduction

Chronic hepatitis C virus (HCV) infection is widely recognized not only for its hepatic complications (1–3) but also for extrahepatic manifestations, including significant neuropsychiatric burden, that clinically manifests with neurological pathologies, behavioral alterations and overt psychiatric conditions (4). In fact, individuals with HCV frequently suffer from psychiatric and cognitive symptoms, including depression, anxiety, fatigue, and impaired quality of life (QoL) (5–7). Studies have consistently demonstrated that HCV-infected individuals experience higher rates of depression (prevalence 15-30%), anxiety disorders (10-25%), and cognitive impairment compared to the general population (8–10). This psychiatric burden appears independent of liver disease severity, suggesting direct viral neurotropic effects (11). In addition, being affected by a chronic infectious disease represents for many patients a discriminating factor and/or a limit to social and personal relationships (12–14). The social stigma associated with HCV infection, often linked to its transmission routes, further compounds psychological distress and contributes to social isolation, relationship difficulties, and reduced treatment-seeking behavior (13, 15, 16). All these factors contribute to a complex clinical profile that demands comprehensive management beyond liver disease itself.

Emerging evidence suggests that psychiatric manifestations in various chronic viral infections or dermatological diseases are not merely consequences of psychosocial stress but may also reflect biological mechanisms directly related to neuroinflammation and immune dysregulation (17–19). In fact, evidence from neuroimaging and cerebrospinal fluid studies demonstrates that HCV can directly invade the central nervous system, where it replicates in astrocytes and microglia, inducing local neuroinflammation (6, 20–22). This process involves dysregulation of neurotransmitter systems, particularly serotonergic and dopaminergic pathways, and persistent elevation of proinflammatory cytokines such as IL-1β, IL-6, and TNF-α (23–25). Moreover, psychiatric and infectious comorbidities are increasingly recognized as bidirectionally linked: chronic viral infections may exacerbate psychiatric vulnerability, while mental disorders may impair immune function and treatment adherence, ultimately worsening medical outcomes (17). These mechanisms may help explain why mental disorders can negatively influence the course of chronic infectious diseases, reduce treatment response, and worsen overall prognosis (17, 26, 27).

The advent of direct-acting antiviral agents (DAAs) has transformed HCV care, offering highly effective therapies with cure rates exceeding 95% and a markedly improved safety profile (28–30). Despite this therapeutic breakthrough, the longer-term neuropsychiatric outcomes following DAA treatment remain incompletely understood, with most research to date limited to short-term follow-up periods. Early evidence suggests that DAAs are generally safe from a psychiatric perspective and may even improve mood and coping, particularly in patients without prior psychiatric history (31–33). However, such findings derive mainly from short-term observation or Patient-Reported Outcomes (PRO) (34), leaving a crucial knowledge gap regarding sustained psychiatric safety and QoL outcomes. Furthermore, while the short-term psychological benefits are evident, it is not yet clear whether these are maintained over time and how they relate to the trajectory of physical QoL, which could be influenced by residual comorbidities and the natural aging process even after viral eradication. In our previously published prospective observational study (35), we evaluated 62 HCV-infected patients at baseline (T0) and 12 weeks after completion of DAA therapy (T1), demonstrating that DAAs did not worsen psychiatric symptoms and were associated with improvements in mood and QoL, especially in patients without psychiatric comorbidity.

Nevertheless, it is not yet known how long these effects last during long-term follow-up, considering both the severity of liver impairment at the time of treatment with DAA and the possible onset of physiological phenomena of aging that could affect QoL. The present study addresses this gap through a long-term follow-up assessment (80 months), in the same cohort.

In particular, the present study aims at elucidating whether the psychiatric and QoL improvements observed after DAA therapy persisted or changed over time at long-term follow-up and to explore differential trajectories based on the presence or absence of a psychiatric history. As a secondary objective, we investigated how coping strategies at baseline could influence long-term changes in psychopathological and quality-of-life outcomes. This investigation provides novel insights into the enduring psychiatric safety profile and quality-of-life outcomes following successful DAA therapy, underscoring implications for integrated psychiatric and hepatological care in HCV management.

## 2 Materials and methods

### 2.1 Study design

This is a prospective longitudinal follow-up study of patients originally enrolled in a prospective observational trial conducted in the Departments of Internal Medicine and Psychiatry, University of Campania “Luigi Vanvitelli”, Naples, Italy. Patients were evaluated at three time points: 1) baseline, prior to starting DAA therapy (T0); 2) 12 weeks after completion of DAA therapy (T1); and 3) long-term follow-up, approximately 80 ± 10 months after DAA initiation.

### 2.2 Participants

Eligible participants for the present follow-up study were (1) adult patients (aged ≥ 18 years), (2) with chronic hepatitis C or HCV-related cirrhosis, (3) who obtained sustained virological response (SVR) after DAA treatment, (4) had undergone psychiatric and clinical assessments at both baseline (T0) and 12 weeks post-treatment (T1) in the previous study (35), and (5) had provided informed consent to be re-evaluated at the long-term follow-up. Exclusion criteria included: (1) withdrawal of consent for participation at follow-up; (2) development of severe medical comorbidities that could independently affect psychiatric outcomes (e.g., stroke, dementia, active malignancy); (3) initiation of new psychiatric medications during the follow-up period; and (4) substance use relapse requiring treatment.

Moreover, following the design of the original study, participants were stratified into two groups: 1) patients with a current or lifetime psychiatric history (Group P); and 2) patients without any psychiatric history (Group NP).

### 2.3 Psychopathological assessment

All patients enrolled in the present study underwent a new psychological evaluation at the same time as the hepatological control visit. The following questionnaire were used in their validated Italian form:

- Hamilton Rating Scale for Depression (HAM-D), to assess the severity of depressive symptoms across several domains, including mood, guilt, insomnia, somatic symptoms, anxiety, and weight loss; each item is rated on a three- or five-point Likert scale with higher scores indicating severe depression symptoms (36);
- Hamilton Anxiety Rating Scale (HAM-A), to evaluate both psychic (mental agitation, tension, fears, and cognitive aspects) and somatic anxiety (physical complaints such as cardiovascular, gastrointestinal or respiratory symptoms; each item is scored from 0 (not present) to 4 (very severe), yielding a total score ranging from 0 to 56. A higher total score indicates more anxiety symptoms (37);
- Symptom Checklist-90-Revised (SCL-90-R), a self-report symptom inventory comprising 90 items for screening and detecting clinical symptoms or indicators of psychological distress; each item is rated on a five-point Likert scale from 0 (“not at all”) to 4 (“extremely”), producing nine primary symptom dimensions: somatization, obsessive-compulsive, interpersonal sensitivity, depression, anxiety, hostility, phobic anxiety, paranoid ideation, and psychoticism. In addition, the Global Severity Index (GSI) reflects the overall level of psychological distress, and higher scores indicate greater psychological distress and symptom severity (38).

### 2.4 Coping strategies and quality of life evaluation

The Coping Orientation to Problems Experienced (COPE) Inventory (39) and the Short Form 36 (SF-36) Health Survey (40) had been used (41). The COPE consists of 60 items grouped into 16 scales, corresponding to distinct ways of problem-solving or modulating emotions. According to the original factor structure proposed by Carver and colleagues, these subscales can be grouped into three broad dimensions: problem-focused coping, emotion-focused coping, and avoidant (or maladaptive) coping. Problem-focused coping includes strategies aimed at directly addressing the source of stress, such as active coping, planning, suppression of competing activities, restraint coping, and seeking instrumental social support. Emotion-focused coping encompasses strategies intended to regulate the emotional response to stress, including seeking emotional support, positive reinterpretation and growth, acceptance, turning to religion, and humor. Finally, avoidant or maladaptive coping reflects disengagement or denial of the stressor, and includes denial, behavioral and mental disengagement, substance use, and venting of emotions.

The SF-36 consists of 36 items that assess perceived health status across eight domains. These domains can be summarized into two higher-order composite scores: the Physical Component Summary (PCS) and the Mental Component Summary (MCS). The PCS mainly represent an overall index of physical well-being and functional capacity. The MCS, on the other hand, primarily serves as a measure of psychological well-being and emotional adjustment. Higher scores on either the PCS or the MCS indicate a better health-related quality of life, whereas lower scores are suggestive of physical or psychological impairment.

### 2.5 Statistical analysis

Before performing the analyses, the distribution of continuous variables was assessed using the Kolmogorov–Smirnov test. Given the small sample size at follow-up and the non-normal distribution of most variables, non-parametric tests were applied throughout the analyses. Within-group longitudinal changes between baseline (T0) and long-term follow-up were evaluated using Wilcoxon signed-rank tests, while Mann–Whitney U tests were used for between-group comparisons (Group P: patients with psychiatric history; Group NP: patients without psychiatric history). Categorical variables were compared using chi-square tests.

Analyses were conducted following a hierarchical approach, with primary focus on within-group longitudinal changes and between-group comparisons. To further explore potential predictors of long-term outcomes, exploratory correlation and regression analyses were conducted. Spearman’s rho correlations were computed between coping strategies and changes over time in psychopathological scores (expressed as Δ). The term Δ (delta) indicates the change between baseline (T0) and long-term follow-up, calculated as Δ = score at follow-up – score at baseline, where negative values reflect symptom improvement (score reduction over time). Subsequently, exploratory multiple regression models were tested to identify predictors of long-term changes (Δ HAM-D, Δ HAM-A, Δ SF-36-PCS, Δ SF-36-MCS, Δ SCL-90-R total score). Independent variables included age, sex, psychiatric history, and the three aggregated coping dimensions (problem-focused, emotion-focused, and avoidant coping). Given the exploratory nature of the analyses and the relatively small sample size, no formal correction for multiple testing was applied, therefore, the results should be interpreted with caution.

An attrition analysis was also conducted to compare sociodemographic and clinical characteristics at baseline (T0) between participants who completed follow-up and patients who were lost to follow-up, in order to assess potential selection biases. Categorical variables were compared using chi-square tests, while continuous variables were analyzed using Mann-Whitney U tests.

Analyses were conducted on complete cases only; no imputation procedures were applied for missing data. A p-value ≤ 0.05 was considered statistically significant. Continuous variables are presented as median values (minimum–maximum), and categorical variables as absolute frequencies and percentages. All analyses were performed using IBM SPSS Statistics, version 30 for macOS, and graphical representations were created with R software (version 4.3.0) using the ggplot2 package.

Given the small sample size at follow-up, internal consistency indices (e.g., Cronbach’s alpha) were not computed, as such estimates may be unstable and difficult to interpret; however, all instruments used have demonstrated robust psychometric properties in previous validation studies, including Italian populations.

### 2.6 Ethics

The study was conducted in accordance with the principles of the Declaration of Helsinki and was approved by the Ethics Committee of the University of Campania “Luigi Vanvitelli” (protocol number 662/2017; AOC/0002372/2024). All participants provided written informed consent at baseline and confirmed their willingness to participate in the long-term follow-up assessment. Patient data were processed in anonymized form to ensure confidentiality and privacy throughout all stages of data collection and analysis.

## 3 Results

### 3.1 Sample characteristics and attrition

While the original study enrolled 62 patients with chronic HCV infection, 24 patients (38.7%) participated in the follow-up evaluation. All patients maintained their SVR. As shown in **Table 1**, 38 patients (61.3%) were lost, of whom 29 (46.8%) were no longer followed at the recruiting center, 6 (9.7%) died (3 for liver-related cause), and 3 (4.8%) refused participation at follow-up. An attrition analysis was performed to compare baseline characteristics between participants who completed the follow-up and those lost to follow-up. No significant between-group differences were found for age, sex, education, employment status, marital status, liver fibrosis or the majority of baseline psychometric variables (p > 0.05). However, patients retained at follow-up showed significantly higher baseline scores on the SCL-90-R Paranoid Ideation subscale (median = 2.5, range = 0-12; mean rank = 37.94) compared with those lost to follow-up (median = 3, range = 0-9; mean rank = 27.43; p = 0.023). In addition, the COPE Mental Disengagement subscale was significantly higher among participants who completed follow-up (median = 9, range = 6-16; mean rank = 39.21) than among those lost to follow-up (median = 8, range = 4-12; mean rank = 26.63; p = 0.007). These findings suggests that, apart from these two dimensions, attrition was not systematically associated with baseline sociodemographic or clinical variables.

**Table 1:** Drop rate.

Total sample at T0 and T1	62
Number of dropouts	38 (61.29%)
*Lost*	29 (46.77%)
*Dead*	6 (9.68%)
*Refused follow-up*	3 (4.84%)
Total sample at follow-up	24 (38.71%)

**Table 2** shows the sociodemographic characteristics of the sample consisting of the 24 subjects who participated in the follow-up study. The sample consisted predominantly of female participants (62.5%), aged between 52 and 86 years, with a median education of 12.5 years (range 5–19). The majority of participants were employed (62.5%), while 25% were retired. Nearly all participants reported having a partner (20 out of 24 subjects). At the time of enrollment, 21.9% of participants had a fibrosis score of zero, 24.4% a score of one, 17.2% a score of two, 25% of participants had a fibrosis score of four, and the remaining 12.5% a score of three.

**Table 2:** General characteristics of enrolled chronic hepatitis C (CHC) patients with (group P) and without (group NP) a current or lifetime psychiatric history.

Variables	Total sample (n = 24)	Group P (n = 11)	Group NP (n = 13)
Gender, male	9 (37.5%)	3 (27.3%)	6 (46.2%)
Age at follow-up, years (median [range])	65 (52-86)	64 (52-84)	66 (53-86)
Education, years (median [range])	12.5 (5-19)	12 (5-17)	13 (5-19)
Employment			
Employed	15 (62.5%)	6 (54.5%)	9 (69.2%)
Unemployed	3 (12.5%)	2 (18.2%)	1 (7.7%)
Retired	6 (25%)	3 (27.3%)	3 (23.1%)
Marital status			
Partnered	20 (83.3%)	9 (81.8%)	11 (84.6%)
Unpartnered (single, divorced, separated, or widowed)	4 (16.7%)	2 (18.2%)	2 (15.4%)
Liver Fibrosis Score at baseline			
0	14 (21.9%)	6 (27.3%)	8 (19%)
1	15 (24.4%)	7 (31.8%)	8 (19%)
2	11 (17.2%)	3 (13.6%)	8 (19%)
3	8 (12.5%)	3 (13.6%)	5 (11.9%)
4	16 (25%)	3 (13.6%)	13 (31%)
Time since DAA initiation, months (median [range])	80 (70-89)	80 (70-89)	79 (72-87)

Data are reported as frequency (%) for categorical variables and as median (range: min–max) for continuous variables.

Throughout the follow-up period, no enrolled patient experienced psychiatric symptoms of sufficient severity to require inpatient psychiatric admission or initiation/change of psychotropic medication. Furthermore, no cases of HCV reinfection were detected during the observation period.

**Figure 1:**
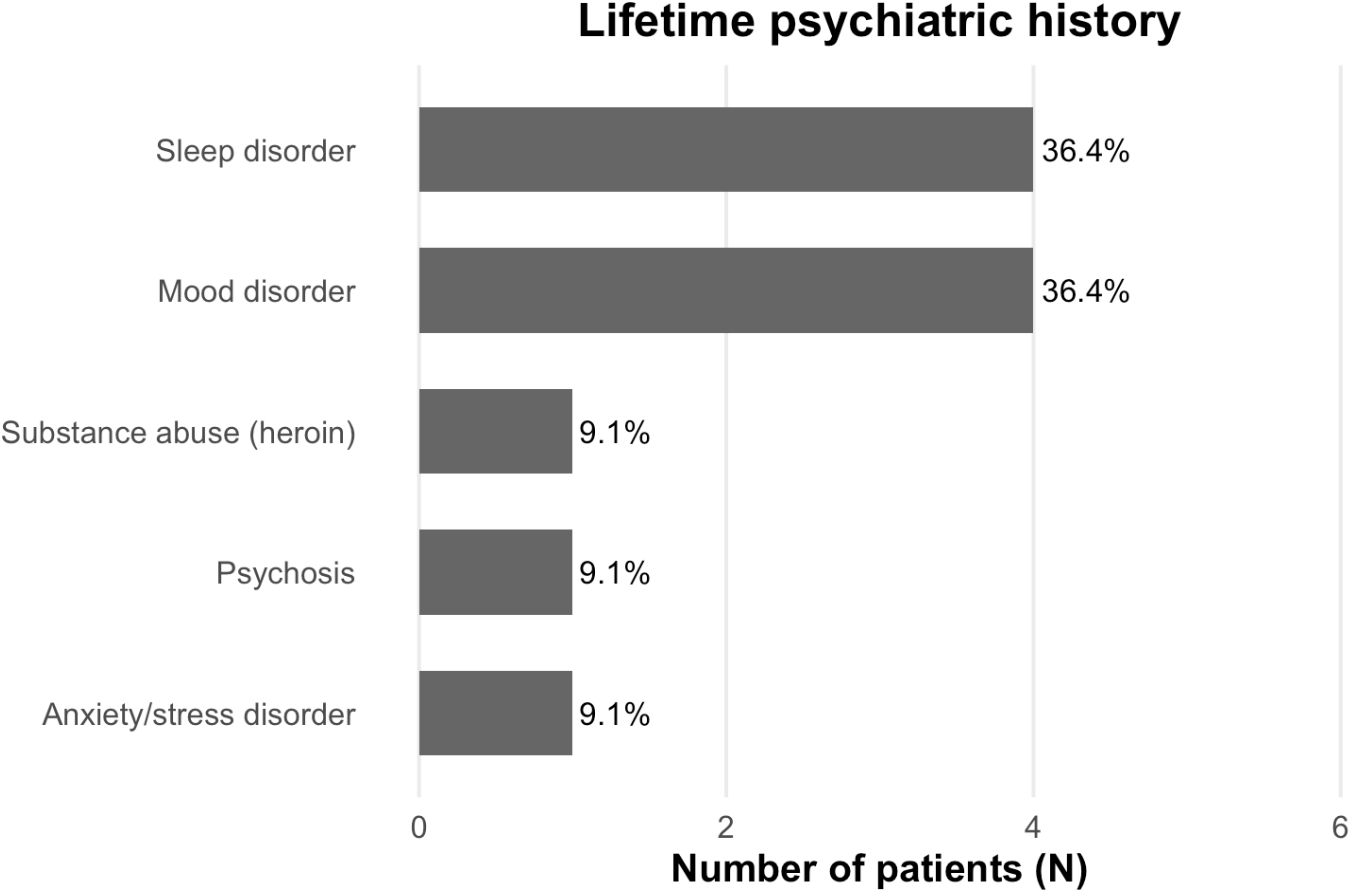
Lifetime psychiatric history of patients in group P.

Following the model of the original study, the sample was further divided into two groups based on the presence (Group P) or absence (Group NP) of a positive personal psychiatric history. **Figure 1** depicts the lifetime psychiatric history of patients in Group P. The Mann–Whitney test for continuous variables and the chi-square test for categorical variables did not reveal any statistically significant differences between the two groups. In other words, no significant sociodemographic differences were found between groups in terms of age, gender, education, employment status, or marital status.

### 3.2 Longitudinal changes within groups (T0 vs. follow-up)

**Table 3** summarizes the clinical characteristics of the two groups for each study time. Specifically, a nonparametric test for paired samples (Wilcoxon Test) was conducted to identify statistically significant differences between T0 and follow-up in the Group P and Group NP.

**Table 3:** Median scores and ranges (min–max) of psychopathological scales in CHC patients with (Group P) and without (Group NP) a current or lifetime psychiatric history, at baseline (T0), after 12 weeks treatment with direct-acting antiviral (DAA) agents (T1), and after 80 (± 10) months follow-up.

Variables	Group P			Group NP		
	T0	T1^#^	Follow-up	T0	T1^#^	Follow-up
HAM-A	15 (4-24)	5 (0-22)	4 (0-25)^**^	8 (5-18)	4 (0-11)	4 (3-19)^***^
HAM-D	16 (2-24)	4 (0-23)	3 (0-25)^*^	7 (3-18)	3 (0-6)	4 (2-13)^**^
SCL-90-R Total score	72 (1-106)	57.5 (1-115)	26 (10-148)	21 (6-107)	22.5 (2-97)	21 (6-124)
SCL-90-R Subscales						
Somatization	9 (0-17)	10 (1-24)	6 (0-26)	4 (0-15)	4 (0-23)	4 (0-32)
Obsessive-Compulsive	10 (0-19)	6 (0-15)	6 (0-22)	5 (1-15)	3.5 (0-14)	3.5 (0-14)
Interpersonal Sensitivity	6 (0-14)	4 (0-12)	2 (0-15)^*^	2 (0-15)	2 (0-9)	1.5 (0-7)
Depression	13 (0-26)	10 (0-27)	5 (0-32)	4 (0-23)	3 (0-8)	4 (0-17)
Anxiety	10 (0-17)	4 (0-18)	3 (0-18)	2 (0-12)	2 (1-11)	1.5 (0-12)
Anger-Hostility	3 (0-11)	4 (0-10)	1 (0-8)	1 (0-5)	1 (0-8)	1 (0-4)
Phobic Anxiety	0 (0-10)	0.5 (0-7)	0 (0-12)	0 (0-8)	0 (0-6)	0 (0-10)
Paranoid Ideation	3 (0-9)	2 (0-10)	1 (0-9)^*^	2 (0-10)	3 (0-12)	1 (0-6)
Psychoticism	5 (1-11)	2 (0-10)	1 (0-8)^*^	1 (0-5)	0 (0-6)	0 (0-9)

Wilcoxon’s Test T0 vs. follow-up: *p < 0.05; **p < 0.01; ***p < 0.001.

HAM-A, Hamilton Anxiety Rating Scale; HAM-D ,Hamilton Rating Scale for Depression; SCL-90-R, Symptom Checklist-90-Revised.

^#^ Data from T1 were included primarily for descriptive purposes to illustrate the trajectory of changes over time, while inferential analyses focused on comparisons between baseline (T0) and long-term follow-up.

In Group P, significant long-term improvements were observed from T0 to follow-up in depressive symptoms as measured by HAM-D (16 vs. 3, p < 0.01) and anxiety symptoms as measured by HAM-A (15 vs. 4, p < 0.01). No statistically significant differences were reported between T0 and follow-up in Group P regarding the SCL-90-R Total score; however, statistically significant differences were found for the subscales of SCL-90-R Interpersonal sensitivity (6 vs. 2, p < 0.05), paranoid ideation (3 vs. 1, p < 0.05), and Psychoticism (5 vs. 1, p < 0.05).

Conversely, SCL-90-R scores in Group NP remained generally stable. The T0 vs. follow-up comparison in scores on the HAM-D and HAM-A scales was statistically significant (7 vs. 4, p < 0.01 and 8 vs. 4, p < 0.001, respectively).

Regarding coping strategies, both groups showed some intra-group changes between T0 and follow-up, as shown in **Table 4**. In patients with psychiatric history (Group P), a significant reduction in active coping (13 vs. 12, p < 0.05), planning 12 vs. 11, p < 0.05) and focus on and venting emotions (13 vs. 9, p < 0.01) subscales was recorded. Reduced scores in active coping (14 vs. 12, p < 0.05), turning to religion (15 vs. 8, p < 0.01) and mental disengagement (9 vs. 7, p < 0.05) subscales were reported in Group NP.

**Table 4:** Median scores and ranges (min–max) of coping orientation to problems experienced (COPE) inventory subscales in CHC patients with (group P) and without (group NP) a current or lifetime psychiatric history, at baseline (T0), after 12 weeks treatment with direct-acting antiviral (DAA) agents (T1), and after 80 (± 10) months follow-up.

Variables	Group P			Group NP		
	T0	T1	Follow-up	T0	T1	Follow-up
Problem-Focused						
Active Coping	13 (10-16)	12 (11-14)	12 (8-14)^*^	14 (12-15)	13 (9-15)	12 (9-15)^*^
Planning	12 (9-16)	12 (8-15)	11 (7-16)^*^	14 (9-16)	13 (11-16)	13 (8-16)
Suppression of Competing Activities	8 (4-12)	12 (6-12)	10 (5-14)	9 (6-15)	11 (9-14)	11 (4-16)
Restraint Coping	10 (7-13)	12 (8-14)	11 (6-15)	12 (7-15)	11 (7-15)	10 (6-15)
Instrumental Social Support	12 (7-15)	12 (9-14)	11(6-14)	14 (5-16)	11 (4-14)	11 (7-15)
Emotion-focused						
Positive Reinterpretation	12 (9-16)	13 (9-15)	11 (4-15)	14 (8-16)	12 (8-15)	13 (9-15)
Acceptance	12 (10-16)	11(10-15)	11 (7-16)	14 (12-16)	13 (8-16)	13 (7-16)
Humor	4 (4-12)	5 (4-10)	7 (4-11)	7 (4-16)	5 (4-11)	5 (4-11)
Turning to Religion	15 (4-16)	12 (4-16)	13 (4-16)	15 (8-16)	14 (4-16)	8 (4-16)^**^
Emotional Social Support	11 (5-15)	12 (7-14)	11 (4-13)	10 (4-16)	9 (4-15)	8 (6-14)
Avoidant or Maladaptive						
Focus on & Venting Emotions	13 (7-16)	12 (7-16)	9 (7-16)^**^	11 (7-16)	10 (6-14)	9 (5-15)
Behavioral Disengagement	6 (4-13)	7 (4-19)	7 (4-13)	8 (4-10)	5 (4-11)	6 (4-10)
Mental Disengagement	9 (6-16)	8 (5-13)	8 (6-9)	9 (8-14)	8 (5-14)	7 (5-14)^*^
Substance Use	4 (4-10)	4 (1-10)	4 (4-17)	4 (4-5)	4 (4-4)	4 (4-11)
Denial	7 (4-12)	6 (4-10)	7 (4-10)	7 (4-11)	4 (4-8)	5 (2-11)

Wilcoxon’s Test T0 vs. follow-up: *p < 0.05; **p < 0.01.

^#^ Data from T1 were included primarily for descriptive purposes to illustrate the trajectory of changes over time, while inferential analyses focused on comparisons between baseline (T0) and long-term follow-up.

Intra-group comparisons of SF-36 subscales between T0 and follow-up are presented in **Table 5**. In Group P, a statistically significant improvement was observed in the Vitality subscale (45 vs. 70, p < 0.05), whereas in Group NP a significant change was detected in the Social Role Functioning subscale (87.5 vs. 50, p < 0.05). No other SF-36 subscales showed statistically significant changes over the 80-month period. In contrast to psychological improvements, a statistically significant decline in the Physical Component Summary (PCS) of SF-36 was observed in both groups: from baseline (T0) to long-term follow-up, the median score dropped from 55.65 to 40.86 (p < 0.05) in Group P and from 51.64 to 39.50 (p < 0.01) in Group NP, indicating a perceived deterioration in physical health.

**Table 5:** Median scores and ranges (min–max) of SF-36 subscales in CHC patients with (group P) and without (group NP) a current or lifetime psychiatric history, at baseline (T0), after 12 weeks treatment with direct-acting antiviral (DAA) agents (T1), and after 80 (± 10) months follow-up.

Variables	Group P			Group NP		
	T0	T1	Follow-up	T0	T1	Follow-up
Vitality	45 (20-65)	55 (25-55)	70 (45-80)^*^	55 (35-85)	67.5 (45-90)	70 (16-90)
Physical Functioning	90 (65-100)	95 (65-100)	75 (15-100)	95 (50-100)	95 (60-100)	82.5 (29-100)
Bodily Pain	80 (32.5-100)	77.5 (32.5-100)	40 (0-100)	100 (22.5-100)	100 (45-100)	35 (0-100)
General Health Perceptions	55 (35-85)	50 (25-65)	55 (45-65)	75 (45-90)	87.5 (40-95)	60 (19-95)
Physical Role Functioning	50 (0-100)	100 (0-100)	100 (0-100)	100 (0-100)	100 (100)	100 (0-100)
Emotional Role Functioning	66.7 (0-100)	100 (0-100)	100 (0-100)	100 (0-100)	100 (100)	100 (6-100)
Social Role Functioning	50 (25-100)	50 (12.5-75)	50 (25-100)	87.5 (62.5-100)	100 (87-100)	50 (4-100)^*^
Mental Health	56 (20-76)	48 (28-64)	64 (20-88)	72 (52-92)	84 (56-92)	80 (23-92)
PCS	55.65 (26-64)	46.14 (35-57)	40.86 (35-58)^*^	51.64 (24-61)	52.82 (33-59)	39.50 (28-58)^**^
MCS	38.46 (14-53)	31.42 (22-52)	49.81 (29-55)	49.72 (18-59)	51.99 (31-59)	52.06 (23-56)

Wilcoxon’s Test T0 vs. follow-up: *p < 0.05; **p < 0.01.

MCS, Mental Component Summary; PCS, Physical Component Summary.

^#^ Data from T1 were included primarily for descriptive purposes to illustrate the trajectory of changes over time, while inferential analyses focused on comparisons between baseline (T0) and long-term follow-up.

### 3.3 Between-group comparisons (psychiatric vs. non-psychiatric history)

Between-group analyses were performed to compare patients with (Group P) and without (Group NP) a current or lifetime psychiatric history across all psychopathological, coping, and quality-of-life variables. Mann–Whitney U tests did not reveal any statistically significant differences between the two groups at the long-term follow-up for any of the examined domains, including psychopathological scales (HAM-D, HAM-A, SCL-90-R subscales), coping strategies (COPE subscales), and QoL measures (SF-36).

These findings suggest that, at approximately 80 months post-treatment, both groups showed comparable psychological profiles and coping patterns.

### 3.4 Exploratory correlation analyses

Spearman’s rho correlations were computed to explore associations between coping strategies at baseline (COPE subscales) and long-term changes in psychopathological outcomes (Δ HAM-D, Δ HAM-A, Δ SCL-90-R subscales, Δ SF-36 subscales).

Several significant associations emerged. Specifically, COPE subscale of Planning correlated positively with Δ Paranoid Ideation (ρ = 0.49, p < 0.05). Suppression of Competing Activities correlated positively with Δ Bodily Pain (ρ = 0.41, p < 0.05) and negatively with Δ Physical Component Summary of SF-36 (ρ = –0.75, p < 0.05).

Restraint Coping showed multiple significant correlations: it was negatively associated with Δ HAMA-A (ρ = –0.41, p < 0.05), Δ Somatization (ρ = –0.44, p < 0.05), Δ Obsessive-Compulsive (ρ = –0.68, p < 0.01), Δ Phobic Anxiety (ρ = –0.44, p < 0.05), and Δ Psychoticism (ρ = –0.52, p < 0.05), while positively correlated with Physical Functioning (ρ = 0.57, p < 0.01), Δ Physical Role Functioning (ρ = 0.50, p < 0.05), Δ Vitality (ρ = 0.48, p < 0.05), and Δ Emotional Role Functioning (ρ = 0.47, p < 0.05).

Instrumental Social Support correlated negatively with Δ Bodily Pain (ρ = –0.70, p < 0.01), Δ General Health (ρ = –0.57, p < 0.01), and Δ Social Role Functioning (ρ = –0.46, p < 0.05), but positively with Δ Paranoid Ideation (ρ = 0.45, p < 0.05).

Acceptance was positively associated with Δ HAMA-A (ρ = 0.45, p < 0.05) and Δ Interpersonal Sensitivity (ρ = 0.44, p < 0.05), and negatively with Δ Vitality (ρ = –0.47, p < 0.05), and Δ Mental Health (ρ = –0.52, p < 0.05).

Focus on and Venting of Emotions correlated negatively with Δ Social Role Functioning (ρ = –0.55, p < 0.05). Behavioral Disengagement showed a negative correlation with HAM-D (ρ = –0.53, p < 0.01). Mental Disengagement correlated positively with Δ MCS (ρ = 0.72, p < 0.05). COPE subscale of Substance Use correlated positively with Δ Vitality (ρ = 0.46, p < 0.05).

No other correlations reached statistical significance (all p > 0.05). Detailed correlation coefficients for all variables are reported in **Table 6**.

**Table 6:** Spearman’s correlations between baseline coping strategies (COPE subscales at T0) and long-term changes (Δ) in psychopathological and quality-of-life measures.

			Δ SF-36										Δ SCL-90-R								
COPE subscales at T0	ΔHAM‐D	ΔHAM‐A	Ph	Ph-RF	BP	GH	Vitality	S-RF	E-RF	MH	PCS	MCS	SOM	OC	SENS	DEP	ANX	HOS	PHOB	PARA	PSY
Active Coping	-0,03	-0,01	0,14	-0,04	-0,05	-0,29	-0,10	-0,04	0,11	-0,27	0,21	-0,33	0,18	0,06	0,14	0,23	0,28	0,17	0,02	0,08	0,23
Planning	0,18	-0,07	0,22	0,10	-0,35	-0,41	0,09	-0,20	0,02	-0,12	0,03	-0,31	-0,15	0,04	0,32	0,27	0,12	-0,01	-0,15	0,49^*^	0,19
Suppression of Competing Activities	0,10	0,17	-0,13	0,18	0,41^*^	0,22	-0,23	0,17	-0,08	-0,21	-0,75^*^	-0,18	-0,07	-0,09	0,01	0,06	-0,06	-0,07	0,05	0,11	0,04
Restraint Coping	-0,33	-0,41^*^	0,57^**^	0,50^*^	0,13	0,18	0,48^*^	0,33	0,47^*^	0,40	0,53	0,33	-0,44^*^	-0,68^**^	-0,25	-0,36	-0,38	-0,19	-0,44^*^	-0,18	-0,52^*^
Instrumental Social Support	0,16	0,00	-0,10	-0,26	-0,70^**^	-0,57^**^	0,12	-0,46^*^	-0,25	-0,07	0,03	-0,27	-0,16	0,08	0,29	0,27	0,04	0,11	-0,14	0,45^*^	0,18
Positive Reinterpretation	0,07	0,00	-0,08	-0,19	0,23	0,18	-0,34	0,09	-0,16	-0,09	0,00	-0,14	-0,04	-0,05	-0,09	-0,13	-0,08	0,01	0,08	0,28	-0,36
Acceptance	0,18	0,45^*^	-0,27	-0,37	-0,18	-0,12	-0,47^*^	-0,18	-0,36	-0,52^*^	-0,19	0,01	0,15	0,14	0,44^*^	0,36	0,29	0,28	0,15	0,26	0,21
Humor	0,09	0,04	-0,06	-0,10	-0,13	-0,11	-0,25	0,15	-0,07	-0,04	0,26	0,05	-0,03	0,13	0,23	0,17	0,13	-0,11	0,16	0,26	0,04
Turning to Religion	-0,33	-0,13	0,32	0,23	0,40	0,20	-0,02	0,22	0,36	-0,18	0,23	-0,37	0,14	-0,02	-0,41	-0,13	-0,10	-0,02	-0,07	-0,29	0,30
Emotional Social Support	0,09	-0,09	0,11	-0,06	-0,09	0,11	0,03	-0,10	-0,15	0,40	0,26	0,19	-0,34	-0,23	0,02	-0,23	-0,29	-0,02	-0,28	0,33	-0,37
Focus on & Venting Emotions	-0,07	-0,19	-0,10	0,10	-0,17	-0,21	-0,14	-0,55^*^	-0,02	-0,26	-0,62	0,08	-0,22	-0,07	0,01	0,06	-0,04	0,07	0,12	0,32	0,09
Behavioral Disengagement	-0,53^**^	-0,27	-0,06	0,06	0,26	0,12	0,16	0,37	0,10	0,16	-0,32	0,21	-0,11	-0,38	-0,41	-0,13	-0,18	-0,16	0,13	-0,16	-0,36
Mental Disengagement	-0,22	-0,19	-0,07	-0,11	-0,11	0,02	0,02	-0,24	0,05	0,08	0,61	0,72^*^	-0,29	-0,30	-0,05	-0,22	-0,17	-0,05	-0,13	-0,20	-0,23
Substance Use	-0,38	-0,28	0,04	-0,03	-0,06	0,08	0,46^*^	0,16	-0,05	0,41	0,28	0,56	-0,25	-0,18	-0,17	-0,14	-0,25	-0,19	-0,19	-0,09	-0,29
Denial	-0,04	-0,06	0,10	0,13	0,07	0,11	-0,01	0,03	0,30	0,08	0,24	-0,47	0,04	-0,06	-0,17	-0,10	0,13	-0,02	0,02	-0,14	0,02

Δ, change from baseline (T0) to long-term follow-up; negative Δ values indicate symptom improvement.

ANX, Anxiety; BP, Bodily Pain; COPE, Coping Orientation to Problems Experienced Inventory; DEP, Depression; E-RF, Emotional Role Functioning; GH, General Health Perceptions; HAM-A, Hamilton Anxiety Rating Scale; HAM-D, Hamilton Rating Scale for Depression; HOS, Anger-Hostility; MCS, SF-36 Mental Component Summary; MH, Mental Health; OC, Obsessive-Compulsive; PCS, SF-36 Physical Component Summary; PARA, Paranoid Ideation; PHOB, Phobic Anxiety; Ph, Physical Functioning; Ph-RF, Physical Role Functioning; PSY, Psychoticism; SCL-90-R, Symptom Checklist-90-Revised; SENS, Interpersonal Sensitivity; SOM, Somatization; S-RF, Social Role Functioning.

*p < 0.05; **p < 0.01.

### 3.5 Exploratory regression analyses

Exploratory multiple regression models were conducted to identify predictors of long-term changes in psychopathological and quality-of-life outcomes. Independent variables included age, sex, psychiatric history, and baseline coping dimensions (problem-focused, emotion-focused, and avoidant). .

**Table 7A:** Multiple regression analysis identifying predictors of long-term change in depressive symptoms (dependent variable: Δ HAM-D) among patients with chronic hepatitis C treated with direct-acting antivirals (DAA).

Independent variables	B	SE	β	*t*	*p*	95% CI, LL	95% CI, UL
Gender, female	3,710	2,624	0,297	1,414	0,175	-1,826	9,246
Age at follow-up	0,206	0,130	0,362	1,591	0,130	-0,067	0,480
Psychiatric history, present	-5,195	2,639	-0,428	-1,968	0,066	-10,762	0,373
Problem-focused coping	-0,323	0,269	-0,304	-1,198	0,247	-0,891	0,246
Emotion-focused coping	-0,097	0,179	-0,113	-0,543	0,594	-0,474	0,280
Avoidant coping	-0,527	0,212	-0,512	-2,483	0,024*	-0,975	-0,079

Adjusted R² = 0.19, p = 0.141.

CI, Confidence interval; HAM-D, Hamilton Rating Scale for Depression; SE, Standard error; LL, Lower limit; UL, Upper limit.

Among all tested regression models, shown in **Tables 7A–E**, only the one with Δ HAM-A as dependent variable reached statistical significance (adjusted R² = 0.30, p < 0.05).

In this model, both psychiatric history (β = –0.47, p = 0.03) and avoidant coping at baseline (β = –0.54, p = 0.01) were significant negative predictors of long-term anxiety change. All other predictors were non-significant (p > 0.80), as showed in **Table 7B**.

**Table 7B:** Multiple regression analysis identifying predictors of long-term change in anxiety symptoms (dependent variable: Δ HAM-A) among patients with chronic hepatitis C treated with direct-acting antivirals (DAA).

Independent variables	B	SE	β	*t*	*p*	95% CI, LL	95% CI, UL
Gender, female	4,583	2,715	0,329	1,688	0,11	-1,146	10,312
Age at follow-up	0,229	0,134	0,361	1,707	0,11	-0,054	0,512
Psychiatric history, present	-6,345	2,731	-0,469	-2,323	0,03*	-12,106	-0,583
Problem-focused coping	-0,515	0,279	-0,436	-1,849	0,08	-1,104	0,073
Emotion-focused coping	0,061	0,185	0,063	0,327	0,75	-0,330	0,451
Avoidant coping	-0,615	0,220	-0,536	-2,798	0,01**	-1,079	-0,151

Adjusted R² = 0.30, p < 0.05.

CI, Confidence interval; HAM-A, Hamilton Anxiety Rating Scale; SE, Standard error; LL, Lower limit; UL, Upper limit.

**Table 7C:** Multiple regression analysis identifying predictors of long-term change in physical health-related quality of life (dependent variable: Δ SF-36-PCS) among patients with chronic hepatitis C treated with direct-acting antivirals (DAA).

Independent variables	B	SE	β	*t*	*p*	95% CI, LL	95% CI, UL
Gender, female	-5,700	19,809	-0,271	-0,288	0,801	-90,933	79,532
Age at follow-up	-0,041	1,288	-0,022	-0,032	0,977	-5,582	5,499
Psychiatric history, present	-0,565	23,200	-0,030	-0,024	0,983	-100,39	99,257
Problem-focused coping	0,112	1,864	0,066	0,060	0,958	-7,909	8,132
Emotion-focused coping	-0,226	0,846	-0,224	-0,267	0,814	-3,865	3,413
Avoidant coping	-0,620	1,859	-0,383	-0,334	0,770	-8,619	7,379

Adjusted R² = -1.96, p = 0.98.

CI, Confidence interval; SF-36 PCS, SF-36 Physical Component Summary; SE, Standard error; LL, Lower limit; UL, Upper limit.

**Table 7D:** Multiple regression analysis identifying predictors of long-term change in mental health-related quality of life (dependent variable: Δ SF-36-MCS) among patients with chronic hepatitis C treated with direct-acting antivirals (DAA).

Independent variables	B	SE	β	*t*	*p*	95% CI, LL	95% CI, UL
Gender, female	-2,673	31,785	-0,068	-0,084	0,941	-139,43	134,088
Age at follow-up	-1,469	2,066	-0,427	-0,711	0,551	-10,358	7,421
Psychiatric history, present	12,353	37,226	0,357	0,332	0,772	-147,82	172,522
Problem-focused coping	-0,611	2,991	-0,192	-0,204	0,857	-13,480	12,259
Emotion-focused coping	-0,044	1,357	-0,023	-0,032	0,977	-5,883	5,795
Avoidant coping	1,312	2,983	0,435	0,440	0,703	-11,523	14,148

Adjusted R² = -1.19, p = 0.91.

CI, Confidence interval; SF-36 MCS, SF-36 Mental Component Summary; SE, Standard error; LL, Lower limit; UL, Upper limit.

**Table 7E:** Multiple regression analysis identifying predictors of long-term change in psychopathological symptoms (dependent variable: Δ SCL-90-R) among patients with chronic hepatitis C treated with direct-acting antivirals (DAA).

Independent variables	B	SE	β	*t*	*p*	95% CI, LL	95% CI, UL
Gender, female	24,709	16,714	0,348	1,478	0,159	-10,723	60,141
Age at follow-up	1,349	0,792	0,428	1,704	0,108	-0,329	3,028
Psychiatric history, present	-16,295	16,546	-0,240	-0,985	0,339	-51,371	18,780
Problem-focused coping	-2,068	1,643	-0,354	-1,259	0,226	-5,552	1,415
Emotion-focused coping	0,407	1,106	0,086	0,368	0,717	-1,937	2,752
Avoidant coping	-2,255	1,337	-0,388	-1,687	0,111	-5,088	0,579

Adjusted R² = 0.04, p = 0.37.

CI, Confidence interval; SCL-90-R, Symptom Checklist-90-Revised; SE, Standard error; LL, Lower limit; UL, Upper limit.

## 4 Discussion

This long-term prospective follow-up study provides novel evidence on the enduring psychiatric and quality-of-life outcomes among patients with chronic HCV infection successfully treated with direct-acting antivirals. At approximately seven years post-viral eradication, our findings indicate that DAA therapy remains its psychiatric safety profile and that psychological benefits observed in the short term are largely sustained, particularly in patients with previous psychiatric morbidity. However, our results reveal a complex long-term trajectory characterized by sustained mental health improvements concurrent with declining physical health-related quality of life, possibly reflecting the intersection of aging, residual comorbidities, and the natural history of post-viral recovery.

In line with our prior 12-week follow-up investigation (35), the present data confirm that DAAs are devoid of detrimental psychiatric effects and may even promote psychological stability in patients with chronic HCV infection. Over an 80-month follow-up, we found significant long-term reductions in depressive and anxiety symptoms, as measured by the Hamilton Rating Scales for depression and anxiety, in both patients with (Group P) and without (Group NP) psychiatric history. These findings suggest that viral clearance may exert durable neuropsychological benefits that extend beyond the early post-treatment period (33, 42).

Interestingly, those with a pre-existing psychiatric history showed notable improvements in specific psychopathological domains of the SCL-90-R, including interpersonal sensitivity, paranoid ideation, and psychoticism. These changes may reflect a progressive attenuation of interpersonal mistrust and suspiciousness that can accompany chronic infectious conditions (13, 43). The persistence of improvement in anxiety and depressive symptoms in this subgroup is clinically relevant, as these patients are typically considered at higher risk for psychiatric instability and treatment non-adherence (44, 45). The data thus reinforce the view that DAA therapy, unlike interferon-based regimens, is not only safe but potentially beneficial for mental health across the psychiatric spectrum.

Group NP patients, those without prior psychiatric illness, maintained psychological stability over time, with continued mild improvement in depression and anxiety scales. The absence of deterioration in this group suggests that the initial psychiatric benefits of DAAs are sustained in the long run and are not merely short-term fluctuations secondary to relief from treatment completion or viral clearance.

Despite the psychological stability observed, both groups exhibited a significant reduction in the Physical Component Summary (PCS) of the SF-36, while the Mental Component Summary (MCS) remained stable or slightly improved. This divergence likely reflects not only the natural course of aging but also the emerging clinical challenge in the DAA era: managing the multimorbidity and potential frailty in an aging cohort of HCV survivors. Eradicating the virus effectively shifts the clinical focus from virology to geriatrics and chronic disease management (46–49). The observed reduction in physical functioning underscores that HCV cure does not equate to a full return to pre-morbid health, and long-term multidisciplinary follow-up remains crucial (50).

However, in Group P vitality significantly improved, suggesting a subjective perception of increased energy and motivation, possibly mediated by reduced depressive and anxiety symptoms. In contrast, Group NP displayed a decrease in social role functioning, which may indicate progressive social disengagement or age-related changes in social activity. This apparent paradox consisting in improved mental health but reduced physical and social vitality has also been described in other long-term studies on HCV survivors (31, 51–53). Such findings highlight the complexity of recovery trajectories after chronic infection and the importance of integrated biopsychosocial care. It should be noted that Group P included patients with heterogeneous psychiatric diagnoses, which may have influenced the variability of outcomes and potentially masked disorder-specific trajectories. Future studies with larger samples should explore these differences in greater detail.

A notable contribution of this study is the longitudinal analysis of coping strategies. Both groups showed a modest but significant reduction in active and planning-oriented coping behaviors, potentially reflecting reduced stress exposure following viral eradication. However, decreases in adaptive strategies such as “active coping” and “planning” might also suggest a shift toward more passive or acceptance-based coping patterns with aging (54).

Correlation analyses revealed intriguing associations between baseline coping styles and long-term psychological outcomes. Restraint coping, characterized by self-control and delayed action until an appropriate time, was consistently related to symptom improvement across several psychopathological domains and to better physical and emotional functioning at follow-up. These results align with prior findings that adaptive and goal-oriented coping predicts psychological resilience and adherence to medical treatment in chronic illness (55).

Conversely, avoidant strategies such as behavioral disengagement were associated with worsening depressive symptoms, supporting the conceptualization of disengagement as a maladaptive pattern linked to poor emotional outcomes (56). Interestingly, higher acceptance at baseline was paradoxically correlated with worse long-term vitality and mental health, suggesting that in the context of HCV-related chronicity, passive acceptance might overlap with resignation rather than adaptive acceptance. Such nuanced interpretations reinforce the need for tailored psychological support focusing on adaptive coping reinforcement, particularly in older adults.

Finally, the regression analysis identified psychiatric history and avoidant coping as significant negative predictors of long-term anxiety improvement. This finding underscores the enduring role of psychological and personality factors in shaping emotional recovery, even years after viral eradication. In practical terms, post–Sustained Virologic Response follow-up protocols could incorporate brief psychological screenings to identify patients with previous psychiatric illness or maladaptive, avoidant coping tendencies. Such individuals may benefit from targeted interventions, such as cognitive-behavioral therapy or mindfulness-based stress reduction, to sustain anxiety reduction, prevent late emotional relapse, and consolidate long-term psychological resilience.

The absence of significant between-group differences at follow-up in psychopathological, coping, or QoL measures suggests that over the years, patients with and without psychiatric history may converge toward a similar psychological profile. This convergence could indicate the normalization of mental health trajectories after viral clearance, reflecting both the biological benefits of reduced systemic inflammation and the psychosocial relief associated with being “cured”. Alternatively, it might also reflect a ceiling effect due to the small sample size and the selective retention of more clinically stable participants at follow-up.

Similarly, the lack of significant changes in many SCL-90-R subscales and COPE dimensions may imply that, after the initial adjustment phase following treatment, coping and symptom patterns stabilize. This stabilization is not necessarily negative: it may reflect long-term equilibrium, where patients adapt to new life circumstances without further psychological fluctuation. Nonetheless, subtle shifts in social functioning and physical vitality highlight the persistence of challenges beyond viral eradication, warranting ongoing psychosocial monitoring.

Our findings extend and partially contrast those from the short-term literature. The positive psychiatric outcomes are consistent with multiple prospective studies reporting improvements in depression, anxiety, and QoL after DAA therapy (34, 53). However, while early studies often reported marked improvements in both physical and mental QoL domains, our long-term results show a more nuanced picture with stabilization or even decline in physical components. This pattern has recently been observed in longitudinal cohorts, where aging, comorbidities, and persistent fatigue contribute to a plateau or decrease in physical QoL despite sustained virologic response (33, 51, 57, 58). Moreover, the absence of new psychiatric symptoms or relapses, even in patients with prior mental disorders, strengthens recent evidence suggesting that DAA regimens are psychiatrically safe across diagnostic subgroups (9, 59). Taken together, these data support a paradigm shift from viewing HCV treatment as potentially destabilizing to considering it as an opportunity for recovery.

This study reinforces the necessity of integrating hepatological and psychiatric care in HCV management, even after viral eradication. The enduring mental health stability observed here may be partly attributed to early psychiatric involvement in treatment pathways, which reduces fear, stigma, and non-adherence (60–62). The persistent improvements in depressive and anxiety symptoms observed in our study may reflect the resolution of HCV-induced neuroinflammation and restoration of neurotransmitter homeostasis. Chronic HCV infection induces a characteristic inflammatory signature involving elevated levels of interferons, interleukins, and chemokines that directly affect brain function. While recent studies indicate that immune dysfunction partially persists after viral clearance, with residual signatures of T-cell exhaustion and altered cytokine profiles (25, 63), the substantial reduction in viral load and associated inflammation appears sufficient to maintain long-term neuropsychiatric benefits. This is consistent with the bio-psycho-neuroimmunological model of HCV-associated psychiatric symptoms, where viral clearance breaks the cycle of chronic immune activation and neuroinflammation (24). Future research using neuroimaging and inflammatory biomarkers could help elucidate the neurobiological correlates of long-term psychiatric outcomes after DAA therapy.

Our findings have several important clinical implications. First, they support the paradigm shift toward viewing DAA therapy not merely as antiviral treatment but as a comprehensive intervention with lasting neuropsychiatric benefits. The sustained improvements in anxiety and depression, particularly in patients with psychiatric history, underscore the importance of integrated hepato-psychiatric care models (35, 51, 57, 64, 65). Second, the divergent trajectories of mental versus physical health-related quality of life highlight the need for tailored long-term follow-up protocols that address both successful aging and residual comorbidities in HCV survivors. Third, the identification of baseline coping strategies as predictors of long-term outcomes suggests potential targets for psychological interventions to optimize post-treatment recovery.

Given the small sample size and natural attrition of long-term cohorts, future multicentric studies should include larger samples and integrate neurocognitive and biological assessments (e.g., markers of systemic inflammation, neurodegeneration, and gut–liver–brain axis function). It would also be valuable to assess whether lifestyle interventions, rehabilitation, or psychological therapy could enhance long-term QoL outcomes in post-HCV populations (66). Finally, as the DAA-treated cohort ages, attention should be directed toward understanding how aging-related psychiatric vulnerabilities (e.g., cognitive decline, apathy, social isolation) intersect with the residual effects of chronic infection and treatment.

### 4.1 Strengths and limitations

This study has several limitations that must be acknowledged, along with noteworthy methodological and conceptual strengths.

First, the sample size was relatively small (n = 24), and the attrition rate high (61%), limiting statistical power and generalizability. The main causes of attrition (relocation, death) are consistent with the extended follow-up duration (~80 months) and the advanced age of the cohort. Second, as patients lost to follow-up might have differed in clinical or psychological characteristics, selection bias cannot be excluded. Third, the absence of a control group of untreated or non-HCV individuals prevents attribution of the observed psychological stability exclusively to DAA therapy. Fourth, while we employed validated psychometric instruments, self-reported measures may be subject to recall and response biases. Fifth, the lack of updated biological markers at follow-up precludes exploration of the mechanistic links between viral clearance and mental health. To mitigate these limitations, we relied on standardized clinical protocols identical to those of the baseline study, used nonparametric statistics appropriate for small samples, and verified that baseline characteristics of retained participants did not differ significantly from those lost to follow-up, except for slightly higher scores in Paranoid Ideation and Mental Disengagement subscales among those who completed follow-up. Nonetheless, replication in larger, prospective, multicenter cohorts is warranted.

Despite these limitations, several strengths distinguish this investigation. Among these: 1) the originality: to our knowledge, this is among the very few studies worldwide to explore psychiatric and quality-of-life outcomes after DAA therapy with a long-term follow-up exceeding six years, addressing a major gap in the literature, which has typically been confined to short-term (12–24 months) observations; 2) methodological rigor: the longitudinal design and the use of non-parametric tests ensured appropriate handling of non-normally distributed data in a small sample, while maintaining statistical robustness. The replication of the same psychometric instruments used at baseline (Fabrazzo et al., 2020) allows for precise temporal comparison across three assessment points; 3) clinical relevance: our findings have direct implications for the post-cure management of HCV patients, highlighting the need for sustained hepatological–psychiatric collaboration and long-term psychosocial follow-up in clinical practice; 4) psychological depth: the inclusion of the COPE Inventory provided a multidimensional understanding of patient adaptation, expanding beyond symptomatology to the underlying psychological mechanisms of resilience and adjustment.

In summary, while limited by sample size and attrition, this study’s novel longitudinal perspective, methodological soundness, and integration of clinical and psychological domains offer valuable insights into the enduring mental health trajectories of DAA-cured HCV patients and lay the groundwork for future multicentric and translational research.

## 5 Conclusion

In conclusion, this long-term prospective follow-up demonstrates that DAA therapy for chronic HCV infection is psychiatrically safe and confers sustained benefits on mood and anxiety symptoms, even nearly seven years after viral clearance. These findings extend our previous results by showing that the early improvements in psychological well-being are largely maintained, irrespective of prior psychiatric history.

However, the decline in physical quality-of-life domains underscores that biological and psychosocial recovery after HCV cure remains incomplete. Integrated long-term follow-up, involving hepatologists, psychiatrists, and rehabilitation specialists, is essential to optimize both physical and psychological outcomes in the post-HCV era. Future studies should clarify the neurobiological and behavioral mechanisms underpinning these trajectories and identify modifiable factors to promote successful aging and mental health maintenance in this growing population of DAA-cured patients.

## Data Availability

The raw data supporting the conclusions of this article will be made available by the authors, without undue reservation.
